# Effects of zirconium element on the microstructure and deuterium retention of W–Zr/Sc_2_O_3_ composites

**DOI:** 10.1038/srep32678

**Published:** 2016-09-06

**Authors:** Hongyu Chen, Laima Luo, Jingbo Chen, Xiang Zan, Xiaoyong Zhu, Qiu Xu, Guangnan Luo, Junling Chen, Yucheng Wu

**Affiliations:** 1 School of Materials Science and Engineering, Hefei University of Technology, Hefei 230009, People’s Republic of China; 2 National–Local Joint Engineering Research Centre of Nonferrous Metals and Processing Technology, Hefei 230009, People’s Republic of China; 3 Research Reactor Institute, Kyoto University, Osaka-fu 590-0494, Japan; 4 Institute of Plasma Physics, Chinese Academy of Sciences, Hefei 230031, People’s Republic of China

## Abstract

Dense W and W–Zr composites reinforced with Sc_2_O_3_ particles were produced through powder metallurgy and subsequent spark plasma sintering (SPS) at 1700 °C and 58 MPa. Results showed that the W–1vol.%Zr/2vol.%Sc_2_O_3_ composites exhibited optimal performance with the best relative density of up to 98.93% and high Vickers microhardness of approximately 583 Hv. The thermal conductivity of W–Zr/Sc_2_O_3_ composites decreased initially and then increased as the Zr content increased. The moderate Zr alloying element could combine well with Sc_2_O_3_ particles and W grains and form a solid solution. However, excess Zr element leads to agglomeration in the grain boundaries. W–1vol.%Zr/2vol.%Sc_2_O_3_ composite had a good deuterium irradiation resistance very closing to pure tungsten compared with the other Zr element contents of composites. Under 500 K, D_2_ retention and release of them were similar to those of commercial tungsten, even lower between 400 K to 450 K. Pre-irradiation with 5 keV-He^+^ ions to a fluence of 1 × 10^21^ He^+^/m^2^ resulted in an increase in deuterium retention (deuterium was implanted after He^+^ irradiation), thereby shifting the desorption peak to a high temperature from 550 K to 650 K for the W–1vol.%Zr/2vol.%Sc_2_O_3_ composite.

Nuclear fusion energy is considered to be the principal way to effectively solve the future energy problem as a clean and infinite energy resource. And it is being developed internationally via the International Thermonuclear Experimental Reactor (ITER) Project, which aims to demonstrate the extended burn of deuterium-tritium (D–T) plasma in a fusion reaction^1^,^2^. The mechanical property of plasma facing materials (PFMs) under D–T plasma irradiation is one of the most important issues for the ITER project. Tungsten (W) and its alloys are primary candidate plasma facing materials for the divertor and the first wall in fusion power reactors because of their high melting point, high thermal conductivity, high strength at elevated temperatures, low sputtering yield in radiation environment and low tritium inventory^3^,^4^,^5^. The shortcomings of pure tungsten and its alloys, such as high temperature, embrittlement problems (e.g., low-temperature brittleness, high-temperature or recrystallization brittleness, and radiation-reduced brittleness and hardness), high ductile-to-brittle transition temperature and low recrystallization, exert a negative influence on their applications and restrict their utilization^6^,^7^,^8^. Thus, developing novel tungsten materials with improved ductility and stability against high temperatures and irradiation properties is of utmost importance.

Many studies have shown that several disperse second-phases particles (e.g., ThO_2_, La_2_O_3_, CeO_2_, Y_2_O_3_, and TiC) can effectively inhibit recrystallization and grain growth as well as improve high-temperature strength and creep resistance by hindering grain boundary (GB) sliding^9^,^10^,^11^,^12^,^13^. Rare earth elements are usually doped into tungsten matrix composites to refine grains, strengthen tungsten grains, increase interface bonding, affect the distribution and morphology of impurities, and so on. The impurities existing in GBs, such as carbon (C), oxygen (O), and nitrogen (N), can seriously affect the wettability between the second-phase particles and tungsten grain/s and reduce the cohesion of GBs. Thus, these impurities are considered one of the main causes of intergranular fracture and exert a significant effect on the fracture toughness of tungsten^14^. Adding small amounts of rare earth elements, such as zirconium (Zr), hafnium (Hf), and tantalum (Ta), can strengthen GBs to a certain degree because these reactive elements bind with impurity elements to form compounds with high melting temperatures^15^,^16^,^17^,^18^. The formed compounds (oxides and carbides) are beneficial to the mechanical property of alloys.

Meanwhile, irradiation embrittlement caused by the implantation of energetic particles (H, D, T, He, and neutrons) into the first wall results in irradiation defects upon fusion reactor operation^19^,^20^. The types of damage for PFMs in a fusion reaction include displacement damage caused by high-energy neutrons and surface damage, such as blistering, erosion and sputtering caused by helium (He) and hydrogen (H) from the plasma^21^. Many studies focused on the irradiation damage of W composites containing irradiation-induced morphological damage and on the underlying mechanisms at the macro level to understand the effect of energetic particles on surface modification of W^22^,^23^,^24^. The mechanism of He bubble formation in W was investigated by A. Debelle *et al.*^25^ and T.F. Yang *et al.*^26^, Their studies revealed that He atoms trapped at the vacancies during implantation form stable vacancy–helium complexes, which tend to form bubbles; the shape of the He bubbles is dominated by surface free energy and elastic free energy. The early stages of nanofuzz growth in tungsten after exposing to 80 eV helium at 1130 °C to a fluence of 4 × 10^24^ He^+^/m^2^ was also studied by C.M. Parish *et al.* Four general grain morphologies were observed: smooth, pyramidal, wavy and terraced, which correlated with the underlying surface normal direction of the grain^27^. The formation mechanism of the H bubble was also studied adequately by W.H. Hu *et al.*^28^, who demonstrated that bubble growth is related to both temperature and irradiation fluence and that internal pressure affects the bubble size. H.B. Zhou *et al.* obtained similar conclusions as those of Hu; they found that defects in materials, such as GBs, dislocations, and vacancies, are the origin for H bubble formation in W^21^. X.S. Kong *et al.* performed a series of first-principles calculations to predict the dissolution and diffusion properties of interstitial hydrogen in tungsten and the influence of temperature and the defect-trapping effect^29^. Hydrogen atoms are extremely difficult to aggregate at interstitial sites to form a stable cluster in tungsten, while helium atoms are energetically favorable to cluster together in a close-packed arrangement between (110) planes forming helium monolayer structure, which was investigated by Yuwei You *et al.*^30^ Given that helium and hydrogen atoms exist simultaneously in a reactor, studying their interaction in irradiation damage is necessary. The presence of He enhances D trapping in the near surface and limits D diffusion into the bulk. By contrast, pre-irradiation of D^+^ ions nearly has no effect on the He retention^31^. However, after a high fluence of 1 × 10^23^ He^+^/m^2^, deuterium retention is reduced in terms of the formation of a linked or interconnected structure of bubbles, which can create an easy release and diffusion path for deuterium desorption^32^.

A previous study showed that Sc_2_O_3_ particles dopant can not only refine the grains and increase the tungsten alloy density but can also improve the strength of the samples^33^. The addition of the Zr element was designed to purify GBs and form compounds with the impurities. The phenomenon of solid solution between the alloying element and tungsten matrix can also improve the strength and ductility of composites. Highly uniform W–Zr/Sc_2_O_3_ powder was prepared in the current study through powder metallurgy and then consolidated through spark plasma sintering (SPS), which can produce fine-grain and high-density materials at a relatively low temperature^34^,^35^. After sintering, the microstructural characteristics and properties of the alloy were characterized.

## Experimental

### Sample preparation

**Figure 1:**
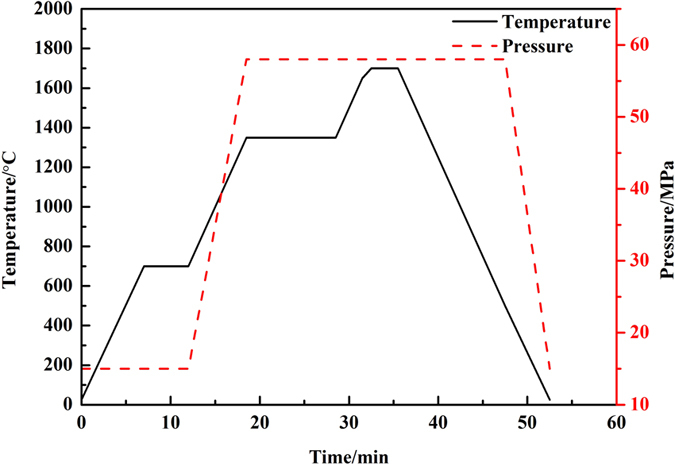
Temperature and pressure variation curves of SPS process for the W–Zr/Sc_2_O_3_ composites.

Three different contents of W–ZrH_2_/Sc_2_O_3_ powder (i.e., W–1vol.%ZrH_2_/2vol.%Sc_2_O_3_, W–3vol.%ZrH_2_/2vol.%Sc_2_O_3_, and W–5vol.%ZrH_2_/2vol.%Sc_2_O_3_) were set to investigate the effects of different contents of Zr alloying element on the microstructure and properties of the composites. An omnibearing ball milling process was utilized to mix the powder in stoichiometric proportions uniformly and to diminish the size of particles under a purified N_2_ atmosphere for 40 h, which seemed to be an appropriate time for both mixing and refining powders. A ball-to-powder weight ratio of 10:1 and a rotation speed of 400 rpm were adopted. Powder collection was conducted in a vacuum glove box with ultimate vacuum of 10 Pa to prevent powders from being exposed to the air as much as possible. The W–ZrH_2_/Sc_2_O_3_ powder was consolidated through SPS (FCT Group, SE–607, Germany) at 1700 °C under 58 MPa pressure for 3 min in Ar + 3% H_2_ atmosphere. Then it was loaded in an electrically and thermally conductive graphite die with a diameter of approximately 20 mm. DC current was applied in pulses together with uniaxial high mechanical pressure during the process. The temperature and pressure profiles of the sintering program in this study are illustrated in Fig. 1. The as-prepared powder specimens were maintained at 700 °C for 5 min to allow ZrH_2_ to decompose completely into Zr and H_2_. Afterward, the powder samples were maintained at 1350 °C  for 10 min to allow Zr and Sc_2_O_3_ to disperse uniformly. Finally, the specimens were heated at 1700 °C for 3 min and then cooled down. The heating and cooling rates in the sintering process were both 100 °C/min. The pressure was increased from 15 Mpa to 58 Mpa whereas the temperature was increased from 700 °C to 1350 °C; both were maintained afterward until the sintering process was over. The samples were approximately 20 mm in diameter and 3 mm in thickness.

### Characterization

The SPS-sintered samples were machined through wire-electrode cutting. The density of the sintered samples was measured with Archimedes method. The relative densities were calculated from the volume fraction. The theoretical densities of W, Zr, and Sc_2_O_3_ were 19.35 g/cm^3^, 6.49 g/cm^3^, and 3.86 g/cm^3^, respectively. The polished sintered samples were subjected to Vickers microhardness testing with MH–3L by measuring from the center to the sample edges with a loading weight of 300 g held for 15 s (the average value was retained after deleting a maximum and minimum value).

A thermal conductivity test was performed with a laser flash thermal analyzer (LFA 457, Germany); the disk samples had 6 mm diameter and 2 mm thickness. Thermal conductivity (λ) was calculated from the thermal diffusivity (α), density (ρ), and specific heat (Cp) through the expression λ = αCpρ. The specific heat capacity was determined with the theoretical rule of mixtures according to the following formula:


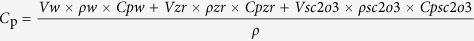


V_W_, V_Zr_, and V_Sc2O3_ are the volume fractions of W, Zr, and Sc_2_O_3_, respectively, whereas C_PW_, C_PZr_, and C_PSc2O3_ are the specific heat values of W, Zr, and Sc_2_O_3_, respectively. The data of C_PW_, C_PZr_, and C_PSc2O3_ at different temperatures were obtained from ref. 36.

The deuterium inventory in the samples was examined through nuclear reaction analysis (NRA) and thermal desorption spectroscopy (TDS). The W–Zr/Sc_2_O_3_ composites were mechanically polished until the surface was mirror-like. These composites were irradiated with D_2_^+^–only with 5 keV in extremely high vacuum and the irradiation dose was 1 × 10^20 ^ions/m^2^, according to the previous experimental results. W–1vol.%Zr/2vol.%Sc_2_O_3_ composites were also exposed to He^+^ ions with a fluence of 1 × 10^21^ He^+^/m^2^ and subsequent D_2_ ions with a fluence of 1 × 10^20^ D_2_^+^/m^2^. After these ion implantations, TDS measurements were performed from room temperature (RT) to 900 K via infrared irradiation with a heating rate of 1 K/s to investigate the D retention behavior. In all cases, the samples were stored in air between the end of exposure and the beginning of the TDS measurement^37^,^38^. Field emission scanning electron microscope (FE–SEM; SU8020, Japan) and energy dispersive X-ray spectroscopy (EDS) with a beam size about 1μm were performed to characterize the microstructures of powder and sintered samples. Transmission electron microscopy (TEM; JEM–2100F, Japan) was also performed to observe the microstructure of the SPS-sintered W–5vol.%Zr/2vol.%Sc_2_O_3_ composite prepared with ion-thinning technology because of its highest second phase content which was easier to observe. Fractured surface was obtained by breaking up the samples at RT artificially. The grain sizes were measured from the SEM images of the fractured surface morphology of the SPS-sintered samples.

## Results and Discussion

### Characterization of powders

**Figure 2:**
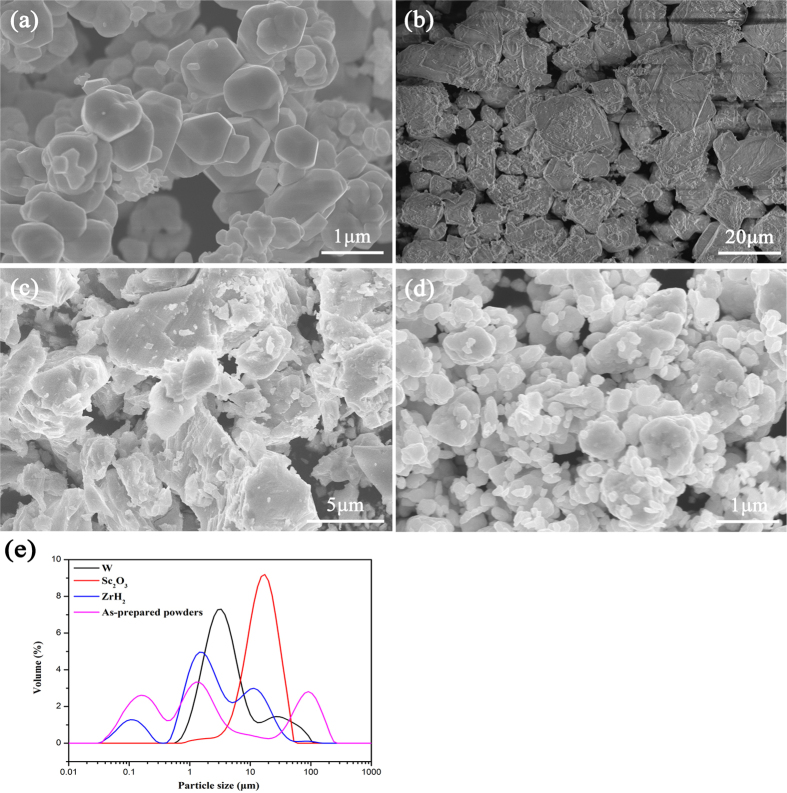
SEM images of the powders: (**a**) W powder; (**b**) Sc_2_O_3_ powder; (**c**) ZrH_2_ powder; (**d**) as–prepared W–ZrH_2_/Sc_2_O_3_ powder and particle size distribution of powders: (**e**).

Before sintering, W, Sc_2_O_3_, ZrH_2_, and the as–prepared powders were observed through FE–SEM equipped with EDS. The different powders shown in Fig. 2. After measurement of Laser Particle Size Analyzer (MS–2000, England), the medium particle diameter of W, Sc_2_O_3_, ZrH_2_, and as–prepared powders were 3.954 μm, 16.699 μm, 2.396 μm, and 1.472 μm, respectively, as shown in Fig. 2e. The width of particle size distribution was also analyzed by formula: (D90-D10)/D50, with values were 6.763, 1.576, 7.085, and 72.51, respectively. The larger the value was, the wider the distribution interval of powders was. After the ball milling for 40 h in a vacuum condition, the average particle size of the mixed powder decreased obviously comparing with the medium particle diameter. However, high-energy ball milling also led to some agglomerations, which was proved by the right side peak of as–prepared powders curve in Fig. 2e.

### Characterization of sintered samples

The relative density, average grain size, and Vickers microhardness of pure W and the samples are shown in Table 1. The addition of the Zr element had a significant influence on the relative density of the W–Zr/Sc_2_O_3_ composites that were all higher than 95%, particularly the W–1vol.%Zr/2vol.%Sc_2_O_3_ samples which exhibited a relative density of 98.93%, as listed in Table 1. The relative density of the W–Zr/Sc_2_O_3_ composites seems to apparently decreased with the addition of Zr. The microhardness of samples was ruleless; it decreased after the first increase with the addition of Zr. The highest average hardness is 614.9 Hv, which corresponded to the W–3vol.%Zr/2vol.%Sc_2_O_3_ samples. The lowest microhardness of 456.1 Hv, which corresponded to the W–5vol.%Zr/2vol.%Sc_2_O_3_ samples, may be attributed to the low relative density and excess Zr, which led to the agglomerations at GBs. The average grain size of the composites was obtained according to the fracture morphologies of the sintered samples. The results demonstrated that the grain size of the samples with different Zr content was inconspicuous at approximately 1–2 μm. Therefore, the grain size of the samples containing much Zr element for oxidation could not be observed with ease.

**Figure 3:**
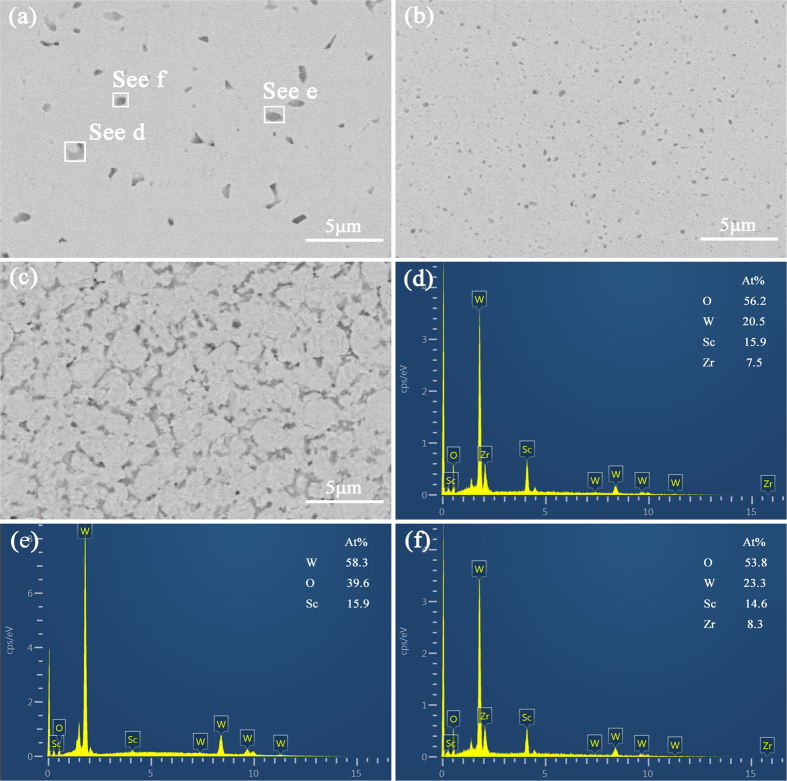
SEM–HA images of sintered samples and EDS spectrums of pane regions in W–1vol.%Zr/2vol.%Sc_2_O_3_ samples: (**a**) W–1vol.%Zr/2vol.%Sc_2_O_3_; (**b**) W–3vol.%Zr/2vol.%Sc_2_O_3_; (**c**) W–5vol.%Zr/2vol.%Sc_2_O_3_ samples; (**d**), (**e**), and (**f**) corresponding EDS spectrums of pane regions in the 1vol.%Zr/2vol.%Sc_2_O_3_ samples.

Figure 3 shows the SEM–HA images of the polished and etched surfaces of the W–1vol.%Zr/2vol.%Sc_2_O_3_, W–3vol.%Zr/2vol.%Sc_2_O_3_, and W–5vol.%Zr/2vol.%Sc_2_O_3_ samples with HA probe and the energy spectrum diagrams of the second-phase particles in the W–1vol.%Zr/2vol.%Sc_2_O_3_ samples. These images confirm that the doped phase could distribute in the samples uniformly. Energy dispersive X-ray spectroscopy was also measured to analyze the black dots qualitatively. The corresponding energy spectrum diagrams of the pane region e and f in Fig. 3a show that mixed phase with W, Sc, and Zr elements also existed in W–1vol.%Zr/2vol.%Sc_2_O_3_ composite, except Sc_2_O_3_ particles. This observation reveals that W, Sc_2_O_3_, and Zr can combine well with one another to form a solid solution. Since the content of Zr in W–1vol.%Zr/2vol.%Sc_2_O_3_ sample is less than W–3vol.%Zr/2vol.%Sc_2_O_3_ and W–5vol.%Zr/2vol.%Sc_2_O_3_ samples, there are still many Sc_2_O_3_ particles exist alone with large particle size. With the increasing of Zr, it can bond well with Sc_2_O_3_ particles and W, form a solid solution and make the size of second phase tinier. However, in the W–5vol.%Zr/2vol.%Sc_2_O_3_ sample, the content of Zr element is excess, which leads to the agglomerations in the GBs.

**Figure 4:**
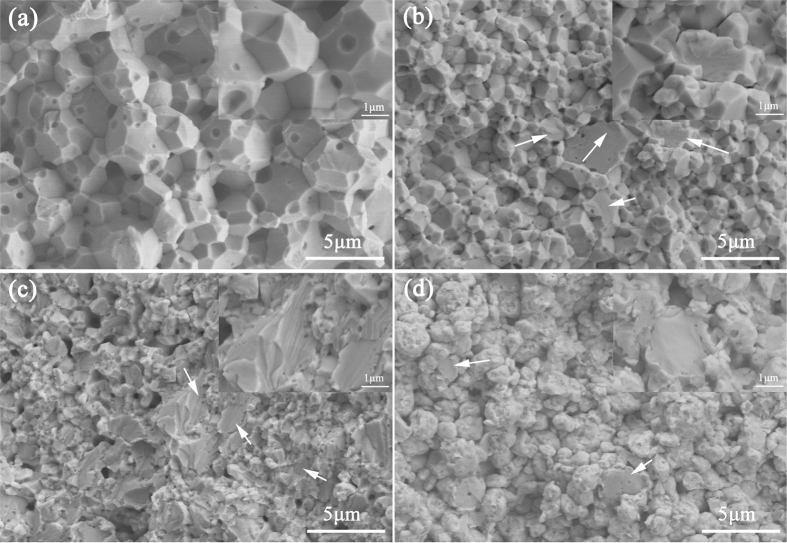
SEM images of fracture surface of (**a**) pure W; (**b**) W–1vol.%Zr/2vol.%Sc_2_O_3_; (**c**) W–3vol.%Zr/2vol.%Sc_2_O_3_; (**d**) W–5vol.%Zr/2vol.%Sc_2_O_3_ samples.

The SEM images of the fracture surface of the sintered composites at RT are presented in Fig. 4. Intergranular fracture appears to be the major fracture for tungsten as a brittle metal. The appearance of pits and shear lips in the fracture surface arrowed in Fig. 4b–d signifies the existence of transgranular fracture, which is apparently a sign of plastic deformation. The high magnification of the transgranular fracture surface is shown in the insets of Fig. 4. The impurities were one of the main causes of intergranular fracture and exerted a significant effect on the fracture toughness of tungsten. The existence and uniform distribution of the Sc_2_O_3_ particles hindered the growth of tungsten grains. The moderate Zr purified the GBs while strengthening the tungsten grains and increasing the interface bonding. Their combination improved the strength and toughness of tungsten to some extent. First-principles calculations also indicated that the cohesion effect of transition metals, like Zr, Hf, and Re on W GBs could significantly strength the GBs^17^. The fracture surface could not be observed conveniently because the exposed Zr oxidized easily in air.

**Figure 5:**
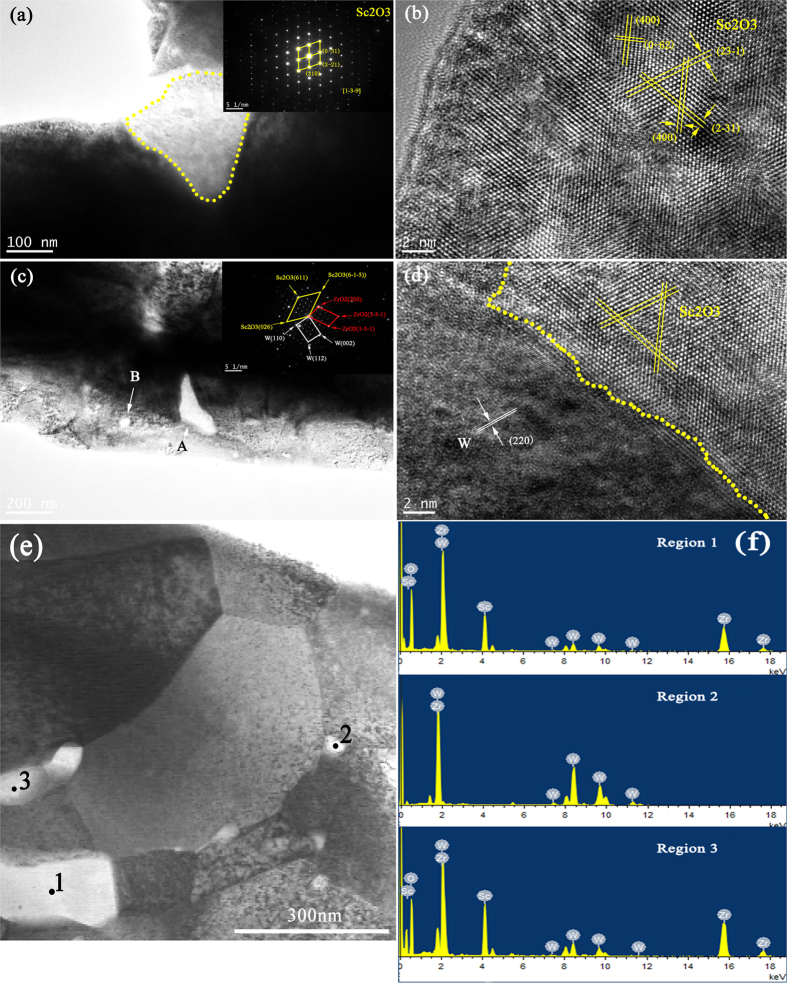
(**a**,**c**,**e**) TEM bright field images of W–5vol.%Zr/2vol.%Sc_2_O_3_ samples and insets of SAED patterns of Sc_2_O_3_ particle and particle A; (**b**,**d**) HRTEM images of the selected regions in (**a**,**c**); (**f**) corresponding EDS spectrums of region 1, 2, and 3 in (**e**).

TEM analysis was performed to characterize the dispersion of the Sc_2_O_3_ dispersed phase and Zr alloying element, as shown in Fig. 5. The TEM bright field images are shown in Fig. 5a,c,e, including the insets of the selected area electron patterns of Sc_2_O_3_ particle and particle A. The interplanar spacing of each phase, as measured from the high resolution TEM (HRTEM) images, corresponded well with the calculated lattice spacing of W (220) and Sc_2_O_3_ (400), (0–62), and (23–1), as shown in Fig. 5b,d. Individual Sc_2_O_3_ particle was still present even though there were excess Zr in the W–5vol.%Zr/2vol.%Sc_2_O_3_ composite. Particle A (Fig. 5c) was a mixed phase, and the SAED pattern measured near the boundary revealed that W, Sc_2_O_3_, and ZrO_2_ existed in all. The Zr element could bond well with both Sc_2_O_3_ particles and W grains, thereby forming a solid solution; this condition corresponded well with the SEM analysis (Fig. 3d). The crystal types of W, Sc_2_O_3_ and ZrO_2_ in the particle A are all cubic. Zr had a high affinity for impurities (such as O, C, and N) in the GBs and could capture these impurities and react to form compounds. Since the compound was ZrO_2_, the impurities were mostly oxygen. Zr alloying element could reduce the O content in the alloy, enhance the cohesion of the GBs, and improve the distribution of elements in the alloy, thereby improving alloy mechanical properties. Other compounds, such ZrC or ZrN were also present theoretically. Some tiny particles existed in the W grains (see particle B in Fig. 5c), which were most likely due to excess Zr element decomposed by ZrH_2_ in the process of sintering. EDS spectrums of Fig. 5f demonstrate that there are W and Zr elements in the nano-sized particles shown in Fig. 5e similar to particle B, which seem to be Zr-W solid solution and Zr_2_W intermetallic phase^17^. The solid solutions similar to particle A of W, Sc_2_O_3_, and Zr also existed in the region 1 and 3 according to their EDS spectrums.

**Figure 6:**
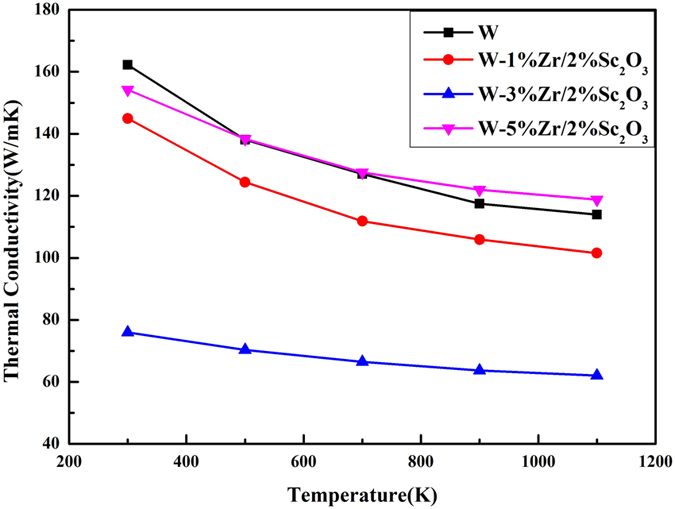
The thermal conductivity of W–Zr/Sc_2_O_3_ composites.

The thermal conductivities of the sintered sample from 300 K to 1100 K were evaluated by comparing them with the thermal conductivity of pure tungsten sintered via SPS at 1700 °C in the previous experiments (Fig. 6). The Sc_2_O_3_ addition decreased the thermal conductivity of the W matrix to some extent, which was attributed to the grain refinement effect of Sc_2_O_3_. The GBs acted as a barrier and impeded thermal conduction. With increasing Zr content, thermal conductivity decreased first and then increased at the same temperature. This observation reveals that the Zr element can bind with the impurity elements (i.e., C, O, and N) in the GBs to form compounds to a certain degree. The formed oxides and carbides strengthened the GBs and hindered thermal conduction, which were the roles of Sc_2_O_3_. It corresponds well with those from the TEM analysis (Fig. 5c). The decreasing trend of thermal conductivities reveals that there are still some impurity elements in the grain boundaries when the content of Zr is 1vol.%. However, the excess Zr element may lead to agglomeration in the GBs and an increase in thermal conductivity for the specific heat values of W and Zr were quite close. These results demonstrate that the thermal conductivity of W–5vol.%Zr/2vol.%Sc_2_O_3_ composite is higher than that of pure tungsten above 700 K.

### Characterization of irradiated W–Zr/Sc_2_O_3_ composites

**Figure 7:**
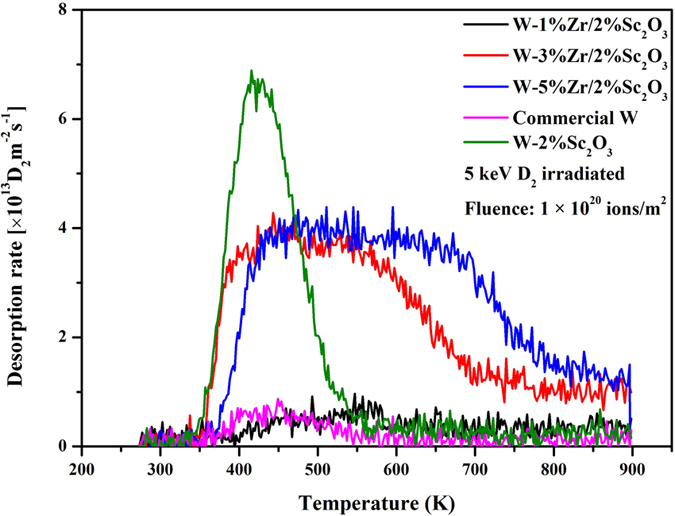
D_2_ thermal desorption for W–Zr/Sc_2_O_3_ composites from 273 K to 900 K with a fixed heating rate of 1 K/s.

The D_2_ TDS spectrum from the W–Zr/Sc_2_O_3_ composites is shown in Fig. 7. According to the displacement threshold energy of 44 eV for tungsten, the minimum energy of hydrogen ions for displacement damage production is calculated to be about 2050 eV^39^. A significant amount of displacements would take place during irradiations with D2 ion of 5 keV, resulting in nucleation and growth of dislocation loops. The defects in the samples, such as dislocation loops, vacancies, GBs, and precipitates, served as trapping sites for deuterium. With the temperature increasing, the loops recover by slipping to surfaces leading to the declining of D_2_ thermal desorption curves. The curve of D_2_ thermal desorption from the W–1vol.%Zr/2vol.%Sc_2_O_3_ composite was relatively stable without an obvious peak in the entire process similar to the curve of commercial W except for a small peak at approximately 550 K. During the temperature range from 300 K to 450 K, the value of thermal desorption from W–1vol.%Zr/2vol.%Sc_2_O_3_ composite is even lower than the commercial W. With temperature increasing, more dislocation loops generated for the second phase would disturb the movement of dislocations, served as trapping sites for deuterium. A large D_2_ peak was observed visibly for the W–2vol.%Sc_2_O_3_ composite without the impact of Zr element at a temperature ranging from 400 K to 500 K, and GBs seem to be the dominant factor. Zr element has a high affinity for impurities (such as O, C, and N) in the GBs and could capture these impurities and react to form compounds, which could purify the GBs, enhance the cohesion of the GBs, and reduce the vacancies of material itself. The solid solution combined with W, Zr, and Sc_2_O_3_ can also enhance the cohesion of particles with tungsten and reduce the vacancies. On the other hand, the relative density of 98.93% of W–1vol.%Zr/2vol.%Sc_2_O_3_ composites also signifies less defects comparing with the 98.6% of W–2vol.%Sc_2_O_3_ composites. Large D_2_ peaks were observed clearly for W–3vol.%Zr/2vol.%Sc_2_O_3_ and W–5vol.%Zr/2vol.%Sc_2_O_3_ composites at a temperature ranging from 400 K to 550 K and 450 K to 700 K, respectively. With the relative density decreased, defects of materials themselves increased served as trapping sites for deuterium, which could generate to defect clusters with temperature increasing and result in the peak of desorption rate shifted to the high temperature side and became higher. Excess Zr element would agglomerate in the GBs according to the SEM analysis, which also leading to the increase of defect clusters such as vacancies and reducing the resistance to radiation. This finding suggests that excessive Zr content is not beneficial for the D_2_ ion irradiation resistance of W alloys. It reveals that moderate Zr can purify the GBs, enhance the cohesion of the GBs, and combine with W and Sc_2_O_3_, therefore reduce the vacancies of material itself, which improves the irradiation property, thereby drastically reduces the peak of desorption rate from W–2vol.%Sc_2_O_3_ samples.

**Figure 8:**
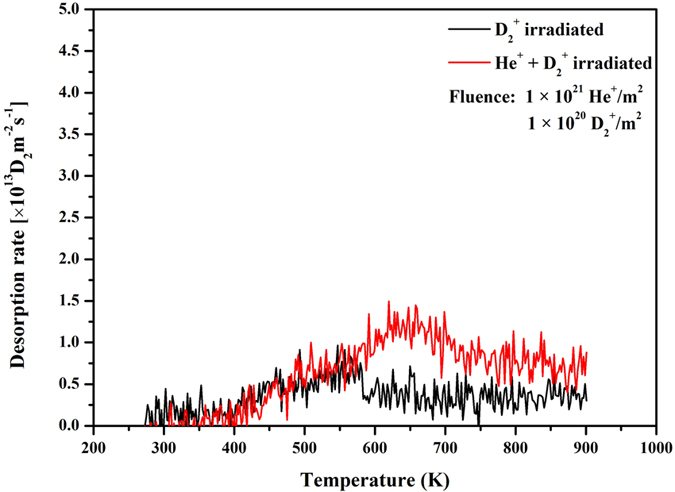
D_2_ thermal desorption for W–1vol.%Zr/2vol.%Sc_2_O_3_ composite and W–1vol.%Zr/2vol.%Sc_2_O_3_ composite pre–irradiated by 5.0 keV helium ions to 1 × 10^21^ He^+^/m^2^.

The deuterium from the samples irradiated with a sequential irradiation of 5 keV-D_2_^+^ ions at RT was measured via TDS to investigate the effects of helium irradiation on deuterium retention properties in W–1vol.%Zr/2vol.%Sc_2_O_3_. The samples were pretreated with He^+^ ions with a fluence of 1 × 10^21^ He^+^/m^2^ and subsequent D_2_^+^ ions with a fluence of 1 × 10^20^ D_2_^+^/m^2^. Figure 8 shows the D_2_ thermal desorption spectra for W–1vol.%Zr/2vol.%Sc_2_O_3_ composite exposed to D_2_^+^ only and He^+^–D_2_^+^. The significant difference in the retention properties between the D_2_^+^ only and He^+^–D_2_^+^ samples was caused by differences in the microstructures of the samples. Only small dislocation loops were formed in the sample irradiated with D^+^ ions, whereas both dislocation loops and bubbles were formed in the sample irradiated with He^+^ ions^32^,^38^. Helium atoms were captured by dislocations and vacancies during implantation and diffusion, thereby forming stable He-vacancy complexes. These complexes were prone to form bubbles as thermally mobile at elevated temperature, which could induce serious volume swelling and embrittlement^39^,^40^. Compared with D_2_^+^ only irradiation, the W–1vol.%Zr/2vol.%Sc_2_O_3_ composites treated with both He^+^ and D_2_^+^ ion irradiation had higher desorption rate from 550 K to 900 K. The D_2_ desorption peak of He^+^–D_2_^+^ samples shifted to a higher temperature from 550 K to 650 K compared with D_2_^+^–only samples. These results demonstrate that pre-implantation of energetic He^+^ ions at RT induced a significant increase in D retention in W. Energetic He^+^ ions can cause structural damage directly through elastic collisions with target atoms^26^. The retained amounts of deuterium gas in the W–Zr/Sc_2_O_3_ samples and Japanese commercial tungsten irradiated by D_2_^+^ only and He^+^–D_2_^+^ are also shown in Table 2. These amounts correspond well with those in the preceding discussion.

## Conclusion

W–Zr/Sc_2_O_3_ composites were sintered through SPS. After sintering, their relative density reached more than 95% and even 98.93%, which was the highest value for the W–1vol.%Zr/Sc_2_O_3_ composite. The microhardness of W–3vol.%Zr/Sc_2_O_3_ was 614.9 Hv, which was greater than that of the other two groups of samples. The grain sizes of composites were generally fine (approximately 1–2 μm) and had a slight difference. The thermal conductivity of the samples decreased as the temperature increased from 300 K to 1100 K. With increasing Zr content, the thermal conductivities of samples initially decreased and then increased. The results revealed that the Zr alloying element can combine well with Sc_2_O_3_ particles and W grains, thereby forming a solid solution. However, excessive Zr element led to agglomerations in the GBs.

The W–1vol.%Zr/2vol.%Sc_2_O_3_ composites possessed optimal mechanical and irradiation properties. The D_2_ retention and release of W–1vol.%Zr/2vol.%Sc_2_O_3_ composites were similar to those of Japanese commercial tungsten exhibiting satisfactory irradiation resistance compared with the other samples. Comparing with the different phenomenon of W–2vol.%Sc_2_O_3_ and W–1vol.%Zr/2vol.%Sc_2_O_3_ composites on deuterium retention properties, GBs seem to be the dominant factor. Moderate Zr element can purify the GBs, enhance the cohesion of the GBs, and combine with W and Sc_2_O_3_, therefore reduce the defects of material itself such as vacancies, which improves the irradiation property. However, pre-irradiation with 5 keV-He^+^ ions to a fluence of 1 × 10^21^ He^+^/m^2^ resulted in an increase in deuterium retention (the deuterium was implanted after He^+^ irradiation). Thus, the desorption peak shifted to a high temperature. This finding demonstrates that pre-irradiation with He^+^ ions can promote D retention by affecting surface topography and by adding dislocation loops and bubbles as trapping sites for deuterium.

## Additional Information

**How to cite this article**: Chen, H. *et al.* Effects of zirconium element on the microstructure and deuterium retention of W–Zr/Sc_2_O_3_ composites. *Sci. Rep.* **6**, 32678; doi: 10.1038/srep32678 (2016).

## Figures and Tables

**Table 1:** Relative density, Vickers microhardness and average grain size of spark plasma sintered W–Zr/Sc_2_O_3_ composites.

Samples	Relative density(%)	Hardness(Hv)	Average grain size(μm)
W	93.50	554.9	3–5
W–1vol.%Zr/2vol.%Sc_2_O_3_	98.93	583.3	1–2
W–3vol.%Zr/2vol.%Sc_2_O_3_	96.77	614.9	1–2
W–5vol.%Zr/2vol.%Sc_2_O_3_	95.00	456.1	1–2.5

**Table 2:** Total deuterium amount retained in the W–Zr/Sc_2_O_3_ samples irradiated by D_2_^+^–only and He^+^–D_2_^+^.

Samples	Total retained deuterium amount
W–1vol. %Zr/2vol.%Sc_2_O_3_(D_2_^+^-only)	2.22 × 10^15^
W–1vol. %Zr/2vol.%Sc_2_O_3_(He^+^-D_2_^+^)	3.79 × 10^15^
W–3vol. %Zr/2vol.%Sc_2_O_3_(D_2_^+^-only)	1.27 × 10^16^
W–5vol. %Zr/2vol.%Sc_2_O_3_(D_2_^+^-only)	1.51 × 10^16^
Commercial tungsten(D_2_^+^-only)	1.40 × 10^15^
W–2vol.%Sc2O3(D_2_^+^-only)	8.10 × 10^15^
